# Novel Variants of *SOX4* in Patients with Intellectual Disability

**DOI:** 10.3390/ijms24043519

**Published:** 2023-02-09

**Authors:** Martin Grosse, Alma Kuechler, Tabib Dabir, Stephanie Spranger, Stefanie Beck-Wödl, Miriam Bertrand, Tobias B. Haack, Corinna Grasemann, Eva Manka, Christel Depienne, Frank J. Kaiser

**Affiliations:** 1 Institute of Human Genetics, University Hospital Essen, University of Duisburg-Essen, 47057 Duisburg, Germany; 2 Northern Ireland Regional Genetics Service, Belfast City Hospital, Belfast BT9 7AB, UK; 3 Limbach Genetics, 28209 Bremen, Germany; 4 Medical Genetics and Applied Genomics, University of Tuebingen, 72076 Tuebingen, Germany; 5 Department of Pediatrics, Faculty of Medicine, Ruhr University of Bochum, 44791 Bochum, Germany; 6 Center for Rare Disease Essen (Essener Zentrum für Seltene Erkrankungen—EZSE), Universitätsmedizin Essen, 45147 Essen, Germany

**Keywords:** *SOX4*, neurodevelopmental delay, intellectual disability, high mobility group (HMG)

## Abstract

SOX4 is a transcription factor with pleiotropic functions required for different developmental processes, such as corticogenesis. As with all SOX proteins, it contains a conserved high mobility group (HMG) and exerts its function via interaction with other transcription factors, such as POU3F2. Recently, pathogenic *SOX4* variants have been identified in several patients who had clinical features overlapping with Coffin–Siris syndrome. In this study, we identified three novel variants in unrelated patients with intellectual disability, two of which were de novo (c.79G>T, p.Glu27*; c.182G>A p.Arg61Gln) and one inherited (c.355C>T, p.His119Tyr). All three variants affected the HMG box and were suspected to influence SOX4 function. We investigated the effects of these variants on transcriptional activation by co-expressing either wildtype (wt) or mutant *SOX4* with its co-activator POU3F2 and measuring their activity in reporter assays. All variants abolished SOX4 activity. While our experiments provide further support for the pathogenicity of *SOX4* loss-of-function (LOF) variants as a cause of syndromic intellectual disability (ID), our results also indicate incomplete penetrance associated with one variant. These findings will improve classification of novel, putatively pathogenic *SOX4* variants.

## 1. Introduction

The SOX family comprises 20 members clustered in eight groups named A to H [1]. SOX proteins have important roles in regulating the development of progenitor cells but also in determining genetic programs in differentiated tissues [2]. SOX proteins mainly function through interactions with a binding partner or by forming heterodimers with other SOX proteins or other transcription factors, or they act as homodimers [2]. For SOX4, syntenin and different POU proteins have been described as possible partners, depending on the target region [3,4,5]. Genetic alterations in *SOX* genes lead to severe congenital disorders covering a broad spectrum of developmental diseases. So far, half of the *SOX* genes have been associated with genetic disorders [6]. SOX4 (OMIM: *184430) is a transcription factor that, together with SOX11 and SOX12, constitutes the SOXC group. They share a common domain called the HMG box that shows nearly 100% identity within this subgroup and about 50% identity with the rest of the SOX family. SOXC proteins have pleiotropic functions and act in concert with each other, as well as in redundancy [5]. *SOX4* is mainly expressed in T and pre-B lymphocytes and in the developing brain. Among other developmental processes, it regulates the inside-out pattern of cortical layer formation via the regulation of *RELN* expression in a redundant manner with SOX11 [7,8,9]. Pathogenic missense variants of SOX genes are mainly clustered within the HMG box, while truncating variants are scattered throughout the gene. Only a few variants “outside” of the HMG domain have been consistently associated with disease so far [6]. Regarding the SOXC group, pathogenic variants of *SOX12* have not been reported yet, while variants of *SOX11* cause a syndrome clinically overlapping with Coffin–Siris syndrome [10]. In addition, de novo variants of *SOX4* have been described as causative for Coffin–Siris syndrome 10 (OMIM: #618506) and, very recently, even more pathogenic variants have been described in 17 patients [8,11]. In addition to that, a homozygous in-frame deletion of eight amino acids within the interdomain region of SOX4 was reported to be associated with features overlapping with these of previously reported patients [12]. Here, we report the identification and functional investigation of three novel *SOX4* variants in patients with developmental delay and intellectual disability.

## 2. Results

**Figure 1:**
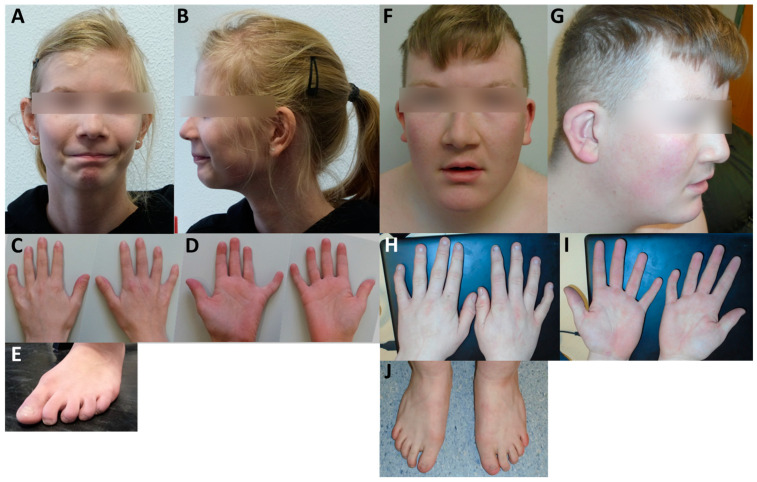
Photographs of patients carrying *SOX4* variants. (**A**–**E**) Patient 1 (c.182G>A; p.Arg61Gln). Her facial features are characterized by bilateral epicanthal folds, a small face, and a broad philtrum. Limb abnormalities include clinodactyly of the fifth finger and small hands and feet. (**F**–**J**) Patient 2 (c.79G>T; p.Glu27*). His facial features are characterized by hypertelorism, epicanthal folds, a smooth philtrum, a thin upper lip, low-set cup-shaped ears, and a prominent forehead. Limb abnormalities include bilateral clinodactyly and hypoplasia of the second and fifth digits.

**Figure 2:**
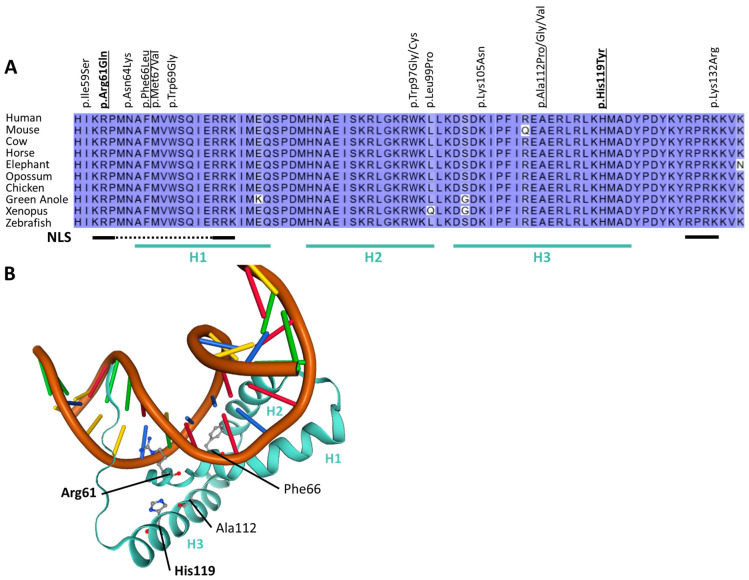
In silico analysis of SOX4 missense variant localization. (**A**) Sequence alignment of the SOX4 HMG-domain of different species. Previously reported missense variants [8,11] and new missense variants from patients 1 and 3 (bold) are indicated. Variants investigated in the luciferase reporter assay are underlined. Sequences reflecting the α-helices and nuclear localization sites are underlined and indicated by H1–H3 and NLS, respectively [13,14]. (**B**) Model of the SOX4 HMG-domain forming a complex with DNA based on template 3u2b [13]. Amino acid residues affected in patients are highlighted. New variants from patients 1 and 3 are shown in bold and α-helices (H) of the HMG-domain are indicated by H1, H2, and H3, respectively.

**Figure 3:**
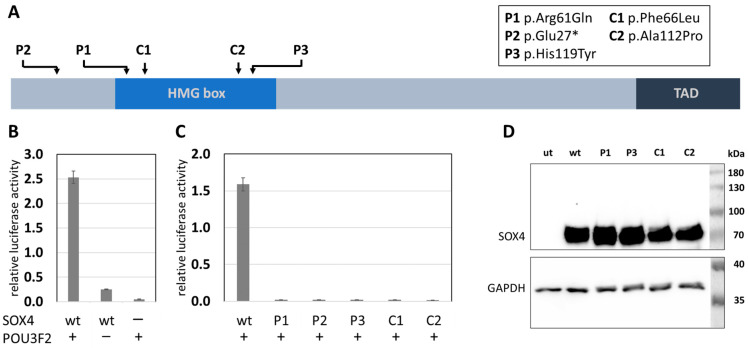
Localization and function of analysed SOX4 variants. (**A**) SOX4 protein structure. Arrows indicate localization of investigated variants. The high mobility group (HMG) box and the transactivation domain (TAD) are indicated. C1 and C2 served as negative controls [8]. The figure is not drawn to scale. (**B**) Activation of luciferase gene expression depends on the interaction of SOX4 and POU3F2. The presence and absence of either SOX4 or POU3F2 are indicated by + and −, respectively. The significance of pairwise differences between co-transfection of SOX4 and POU3F2 constructs and single transfection of either construct was verified for each single experiment using Welch’s unpaired t-test, giving a *p* < 0.02. (**C**) SOX4-mediated activation was abolished by all SOX4 variants. The presence of POU3F2 is indicated by +. Data shown in (**B**,**C**) were normalized for transfection efficiency using constitutive Rluc expression (pRL-TK). Experiments were conducted at least three times independently and the results of one of these experiments are shown. The significance of pairwise differences between the SOX4 wt and either SOX4 variant was verified for each single experiment using Welch’s unpaired t-test, giving a *p* < 0.02. (**D**) Western blot showing proper expression of all SOX4 constructs (SOX4Glu27* excluded). ut, untransfected; wt, wildtype.

**Figure 4:**
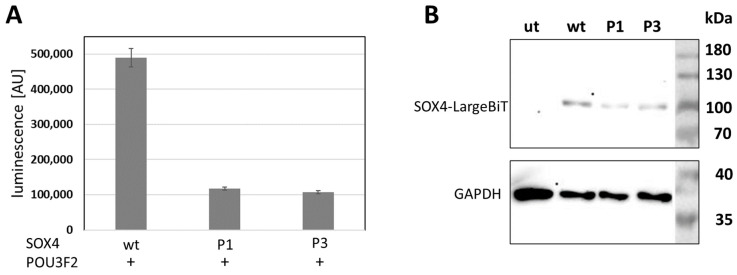
Effects of novel SOX4 variants on protein–protein interaction. (**A**) Novel SOX4 variants reduced SOX4–POU3F2 interaction dramatically. The presence of POU3F2 is indicated by +. Experiments were conducted three times independently and the results of one of these experiments are shown. The significance of pairwise differences between SOX4 wt and either SOX4 variant was verified for each single experiment using Welch’s unpaired t-test, giving a *p* < 0.02. (**B**) Western blot showing the proper expression of the transfected SOX4 constructs. AU, arbitrary unit; wt, wildtype; P1, p.Arg61Gln; P3, p.His119Tyr; ut, untransfected.

Using whole exome sequencing, we identified three novel variants of *SOX4*. Two of those were de novo (patient 1: c.182G>A, p.Arg61Gln, Figure 1A–E; and patient 2: c.79C>T, p.Glu27*, Figure 1F–J), while the third (patient 3: c.355C>T, p.His119Tyr) was inherited from an unaffected mother who was clinically re-evaluated. Phenotypic features of all new patients described here match with those described previously in other patients with variants of *SOX4* and include intellectual disability, developmental delay, and behavioural concerns. In addition, patient 3 suffered from three to four seizures a week, while patients 1 and 2 did not experience recurrent seizures. Heart problems were reported for patients 1 and 2 but were not observed in patient 3. A detailed clinical description of all three patients is given in the Supplementary Materials, and clinical features are provided in Table S2. While variants identified in patients 1 and 3 affected highly conserved amino acids within the first (H1) and third α-helix (H3) of the HMG box (Figure 2A,B), the variant in patient 2 generated a premature stop codon and was predicted to result in a truncated protein lacking the HMG and transactivation domain. Before analysing the SOX4 variants localized at different positions within the HMG box or its N-terminal region (Figure 3A), synergistic interaction of SOX4 and POU3F2 was tested to assure the functionality of our assay. Single expression of wt *SOX4* or *POU3F2* did not significantly affect luciferase expression, whereas co-expression strongly activated reporter gene expression (Figure 3B). This synergistic effect was abolished for all three novel variants (p.Arg61Gln, p.Glu27*, p.His119Tyr), as well as for the two previously reported pathogenic variants (p.Phe66Leu, p.Ala112Pro) included as controls (Figure 3C). We investigated the physical interaction of wt and variant SOX4 as Large BiT fusion proteins with the complimentary POU3F2 as a Small BiT fusion protein. Both novel SOX4 variants reduced the protein–protein interactions of SOX4 and POU3F2 dramatically (Figure 4A). Unspecific protein–protein interactions were excluded by testing combinations of SOX4-Large BiT fusion protein and Small BiT peptide alone or POU3F2-Small BiT fusion protein and Large BiT peptide alone, respectively. We excluded protein instability or insufficient expression as causes of the observed effects by using Western blotting, confirming proper expression of the different *SOX4* constructs except SOX4 p.Glu27*, which lacks the entire HMG domain and was too small for visualisation using SDS mini-gel (Figure 3D and Figure 4B). The weak bands of the *SOX4* constructs shown in Figure 4B were expected and reflected the low-level expression due to the usage of the HSV-TK promoter.

## 3. Discussions

So far, only a few *SOX4* variants have been described as affecting SOX4 function. We contribute to further clarification by reporting three novel *SOX4* variants that resulted in a complete loss of transactivation activity in reporter gene assays. In addition, we provide functional data to show the effects of single amino acid substitutions on the formation of the SOX4 complex with its cofactor POU3F2.

SOX4 contains two functionally important domains; namely, an HMG box and a C-terminal transactivation domain (TAD). The HMG box facilitates DNA binding, bending, and nuclear trafficking [7,15], whereas the latter domain mediates the interaction with different cofactors. Truncated proteins missing the C-terminal TAD are not able to interact with their binding partners, resulting in a loss of their transactivation activity [5]. The variant c.79G>T (p.Glu27*) identified in patient 2 resulted in truncated SOX4, lacking both functional domains. Although early-truncating mutations often result in the absence of a gene product because of nonsense-mediated m-RNA decay (NMD), aberrant transcripts of *SOX4* are not degraded by NMD because *SOX4* is encoded by a single exon [16]. Therefore, the 66 amino acid residues spanning the N-terminus of SOX4 were included in our investigations. It was assumed that SOX4 p.Glu27* would no longer be able to facilitate any of its functions as the HMG domain and the TAD, both known to be crucial, were missing.

The exchange of an arginine at position 61 for glutamine results in a highly conserved residue within HMG domains of different proteins and species [8]. In addition to its location within a predicted bipartite nuclear localization signal [14], previous investigations have provided evidence of its role in DNA binding for Sox4, where it contacts the base pairs T11 and C7 of the *Lama1* enhancer region, as well as other HMG box-containing proteins, such as Sox2, where it is involved in binding the *FGF4* enhancer [13,17]. In addition to its importance for DNA binding, arginine 61 is also critically involved in the interaction between SOX4 and other proteins, such as GATA-3. In particular, binding of Sox4 to GATA-3 was abolished when Arg61 and Pro62 were both replaced by an alanine [18]. A minor contribution of the HMG domain was also shown for the protein–protein interactions of SOX2 and δEF3, a PAX6 homolog in chickens [19]. We also showed that alterations to amino acids within the HMG domain could prevent SOX4 complex formation with POU3F2. The variant identified here resulted in an exchange of a positively charged arginine for a neutral glutamine residue, which prevented SOX4–POU3F2 interaction in vitro and, additionally, might directly influence DNA binding or nuclear import, all of which affect SOX4 function in transcriptional regulation. 

In addition, the second novel missense mutation (p.His119Tyr), which results in the exchange of a highly conserved residue in all SOX proteins of different species, completely abolished SOX4 activity in our reporter assays. For the protein–protein interactions of SOX4 and POU3F2, it was shown that the C-terminal transactivation domain is important, while influence from the HMG domain was not investigated [5]. In contrast to this, it was shown that, for the interaction of Sox2 with its binding partner Pou5f1, the C-terminal region of the HMG domain serves as an interaction platform and that the specific amino acid positions involved differ depending on the target gene. Even though the influence of His101 (corresponding to His119 in SOX4) was not assayed specifically, the surrounding amino acids reduced the ability for Sox2–Pouf51 interaction [17]. However, our results point to the importance of His119 for the interaction of SOX4 and POU3F2, thus indicating the involvement of the corresponding HMG domain region. Interestingly, this variant, identified in patient 3, was also identified in the asymptomatic mother. It is tempting to speculate that this variant might represent the first example of incomplete penetrance for “SOX4-related disorders”, a phenomenon already observed for other disorders caused by variants of *SOX* genes, such as *SOX9*-associated campomelic dysplasia and sex reversal [20,21,22], *SOX11*-associated coloboma [23], and *SOX5*-associated Lamb–Shaffer syndrome [24]. Whether the mother is mosaic for this variant, as another possible explanation of our observations, could not be absolutely excluded, although Sanger sequencing performed on DNA extracted from peripheral blood did not suggest somatic mosaicism in this tissue.

## 4. Material and Methods

Three novel variants of *SOX4* were identified by whole-exome sequencing. Patients 1 and 3 were collected as part of national collaborations and patient 2 was identified via DECIPHER [25], corresponding to number 307135. Exome sequencing was performed at the respective institutions. Referring physicians provided detailed developmental, neurological, and behavioural histories of the patients. Patient information was anonymised before data sharing. Variants were described according to the *SOX4* NM_003107.2 RefSeq transcript.

Wildtype (wt) and mutant (p.Arg61Gln and p.His119Tyr) *SOX4* were amplified from control or patient genomic DNA and inserted into the pcDNA3.1(+)/*myc*-His B expression plasmid. The nonsense variant (p.Glu27*) and two previously reported pathogenic variants (p.Phe66Leu and p.Ala112Pro) [8] used as controls were generated by site-directed in vitro mutagenesis using the Q5 high-fidelity polymerase (Ipswich, MA, USA, New England Biolabs). Sequences of all primers are listed in Table S1. POU3F2 was expressed from pcDNA3.1+/C-(K)DYK-*POU3F2* (OHu10724D, Piscataway, NJ, USA, GenScript Biotech Corporation). A modified *luciferase*-expressing plasmid was generated by inserting three repeats of the *FXO*-fragment containing binding sites for SOX4 and POU3F2 [5] into pGL4.23 (Madison, WI, USA, Promega Corporation). HEK293 cells were seeded in a 24-well plate to obtain around 70 % confluence at the day of transfection. The co-transfection was performed in triplicate using 125 ng of either wt- or mutant *SOX4-*expressing plasmid, together with the same amount of *POU3F2*-expressing plasmid and 250 ng of the luciferase reporter plasmid. One day after transfection, plates were analysed following the protocol for the Dual-Luciferase^®^ Reporter System (Promega Coporation) and signals were detected using a Centro LB 960 luminometer (Bad Wildbad, Germany, Berthold Technologies GmbH & Co. KG). Vectors used for investigation of protein interaction were generated by amplification and insertion of the corresponding wt or mutant fragments from formerly generated expression vectors into modified NanoBiT^®^ (Promega Corporation) expression plasmids containing either FLAG- or myc-tags. HEK293 cells were seeded in a 24-well plate to obtain around 70 % confluence at the day of transfection. A total of 250 ng of either wt or variant *SOX4*-Large BiT-expressing NanoBiT^®^ vector (pBiT1.1-C-myc) was co-transfected with 250 ng of the complementary *POU3F2*-Small-BiT-expressing NanoBiT^®^ vector (pBiT2.1-N-FLAG). Six hours after transfection, each well was split into 3 wells of a 96-well plate. One day after transfection, the plates were analysed following the protocol of the NanoBiT^®^ PPI System (Promega Corporation) and signals were detected using a Centro LB 960 luminometer (Berthold Technologies GmbH & Co. KG). All experiments were conducted at least three times and pairwise significance was verified per each experiment independently using Welch’s unpaired t-test. To verify proper expression of *SOX4* constructs, HEK293 cells were seeded in a six-well plate to obtain a confluence of 70 % at the day of transfection. A total of 2 µg of the corresponding plasmid was transfected and cells were harvested one day after transfection and lysed using RIPA buffer. The cell lysates were separated using a 10 % polyacrylamide gel, and SOX4 variants and GAPDH were detected using an antibody against myc-tag (Danvers, MA, USA, Cell Signaling Technology, #2272) or GAPDH (Cell Signaling Technology, #2118), respectively. 

Sequence comparison of the SOX4 HMG boxes of different species was performed using the Clustal Omega alignment tool available at the UniProt website [26]. A model of the SOX4 HMG box interacting with DNA was generated with Swiss-Model utilizing the 3u2b.1 template [13,27].

## 5. Conclusions

In conclusion, we report three novel pathogenic variants of *SOX4,* all abolishing SOX4 function as a transcription factor in our functional assays. We extended our analyses by including additional known disease-causing variants and further affirm the necessity of in vitro analyses to evaluate the functional relevance of novel *SOX4* variants identified in patients with Coffin–Siris spectrum disorders. By providing new insights into the functional relevance of disease-causing *SOX4* variants, we address the importance of better characterization and classification of the rising numbers of variants obtained from NGS analyses. 

Additionally, we obtained the first indications that incomplete penetrance of a novel *SOX4* variant, affecting a highly conserved residue within the HMG domain, may be a genetic cause of neurodevelopmental delay, further highlighting the need for functional investigations to classify variants of unknown physiological significance identified by whole-exome or whole-genome sequencing analyses.

## Data Availability

The data presented in this study are available in the Supplementary Material.

## Supplementary Materials

The following supporting information can be downloaded at: https://www.mdpi.com/article/10.3390/ijms24043519/s1.
