# Supplementary material for: Novel Variants of SOX4 in Patients with Intellectual Disability
Source: Int J Mol Sci. 2023 Feb 9;24(4):3519. doi: 10.3390/ijms24043519 (PMC9964377; doi:10.3390/ijms24043519)
Supplement: Supplementary file 1 [file ijms-24-03519-s001.zip › ijms-2197151-supplementary.pdf]

Table S1: Sequence of primers and oligos used for plasmid generation. All sequences are written in 5’-3’ direction.

	**Forward oligo**	**Reverse oligo**	**Comment**
**Expression plasmids used for Luciferase Reporter Assay**			
Wt SOX4<br>p.Arg61Gln<br>p.His119Tyr	GGTACCATGGTGCAGCAAACCAACAATG	GGATCCGTAGGTGAAAACCAGGTTGGA	Amplified from genomic DNA
p.Glu27*	Same as above	GGATCCGAGGCCGGCGCCCGAGTCCG	Mutagenesis Primer
p.Phe66Leu	ATGGTGTGGTCGCAGATCGAGCGGC	TAAGGCGTTCATGGGTCGCTTG	Mutagenesis Primer
p.Ala112Pro	CGGAGCGGCTGCGCCTCAAGCAC	GCTCTCGAATGAAAGGGATCTTG	Mutagenesis Primer
***luciferase* expressing plasmid**			
FXO-fragment	TCGAGTAAGTATCCCATTAGCATCCAAACAAAGAGTTTTCTTAAGTATCCCATTAGCATCCAAACAAAGAGTTTTCTTAAGTATCCCATTAGCATCCAAACAAAGAGTTTTCTTAAGTATCCCATTAGCATCCAAACAAAGAGTTTTCTTAAGTATCCCATTAGCATCCAAACAAAGAGTTTTCT	AGCTTAGAAAACTCTTTGTTTGGATGCTAATGGGATACTTAAGAAAACTCTTTGTTTGGATGCTAATGGGATACTTAAGAAAACTCTTTGTTTGGATGCTAATGGGATACTTAAGAAAACTCTTTGTTTGGATGCTAATGGGATACTTAAGAAAACTCTTTGTTTGGATGCTAATGGGATACTTA	Contains five iterations of the FXO fragment, but only clones with three iterations of full length FXO could be selected due to cloning process
**Expression plasmids used for the NanoBit Assay**			
Wt SOX4 and variants	CTCAGAAGAGGATCTGGCTAGCATGGTGCAGCAAACCAACAATG	GGTACCGTCGACTGCAGAATTCgaGTAGGTGAAAACCAGGTTGG	Amplified from corresponding expression plasmids
POU3F2	CTCAGAAGAGGATCTGGCTAGCATGGCGACCGCAGCGTCTAACC	GAATTCGAAGCTTGAGCTCGAGcCTGGACGGGCGTCTGCACCCCGTG	Amplified from corresponding expression plasmid

Detailed clinical description of all three patients

Patient 1

Patient 1 is a 12-year-old girl, born to non-consanguineous Caucasian parents after normal pregnancy at 37+0 weeks of gestation with 2420 g weight (3rd-10th percentile, -1.3 SD), 50 cm length (25th-50th percentile, 0.2 SD) and 33 cm OFC (25th-50th percentile, -0.6 SD) and normal postnatal adaptation (Apgar 10/10). Postnatally, a large anterior fontanelle was observed. The girl showed developmental delay, she started crawling at 12 months and unsupported walking at 19 months. Especially her speech development was significantly delayed, the exact age of first spoken words could not be remembered, at age 2 ½ years she was able to speak only 20 simple words. At age 11 months she suffered a single febrile seizure. Subsequently initiated diagnostic investigations showed normal EEG, echocardiography revealed a small hemodynamically not relevant ASD that closed spontaneously, and a mild dilatation of the aorta ascendens, ECG showed a Wolf-Parkinson-White syndrome without paroxysmal tachycardia. She was also diagnosed with a unilateral doubled kidney. She has normal hearing, she wears glasses because of a refractive error.

Upon the first clinical genetics consultation at age 2 ½ years, she had normal measurements [length 88 cm (10th, -1.4 SD), weight 11 kg (10th-25th percentile), OFC 47.5 cm (10th-25th percentile, -1.4 SD)]. Her facial features were characterised by bilateral epicanthal folds, a small face, and a broad philtrum. She had bilateral fifth finger clinodactyly and small hands and feet. Her sleep is normal. She suffers from chronic constipation. At age 9 years, a thoracic scoliosis was diagnosed and treated with physiotherapy and a brace. IQ tests performed at the of age 8 7/12 years and 10 years revealed scores of 79 and 81, respectively, another test at the age of 12 years confirmed a mild ID (IQ 58). She takes regular medication for a hypothyroidism (L-Thyroxin). She also suffers from an attention deficit hyperactivity disorder that is treated with methylphenidate. At age 11 8/12 she had normal measurements [height 144.5 cm (10th-25th percentile, -0.9 SD), weight 32.8 kg (10th-25th percentile), OFC 52 cm (10th-25th percentile, -1.1 SD)]. Upon clinical evaluation at age 13 years she could speak in whole sentences, was able to read simple single words and to do simple addition and subtraction tasks.

Her facial features (Fig. 1 A - E) were characterised by deep-set eyes, mildly upslanting palpebral fissures, a broad philtrum, and a cupids bow. Her fingers were relatively short with short terminal phalanges and clinodactyly 5.

Conventional karyotyping and array analyses showed normal results. Trio-WES revealed a *de novo* probably pathogenic variant in *SOX4* c.182G>A, p.Arg61Gln, which was absent in both parents.

Patient 2

Patient 2 was first seen in genetics clinic at the age of 12 years with clinical phenotype of moderate learning difficulties, facial dysmorphism, behavioral issues and congenital heart disease. He was born at term. His motor development was age appropriate. He had some speech delay. He was diagnosed to have a small VSD and mild to moderate mitral regurgitation with cleft mitral valve. His facial dysmorphism (see figure 1F - J) consisted of hypertelorism, epicanthal folds, smooth philtrum, thin upper lip, low set cup shaped ears and prominent forehead. He was also noted to have nail hypoplasia and wide spaced nipples. His anthropometric measurements revealed a normal weight (75th centile) and height (25th centile) and a microcephalic head circumference (2nd centile). This was consistent pattern of growth since birth. He underwent bilateral myringotomy and vent insertions for glue ears. He has ADHD and episodes of aggressive and difficult behavior. He is on medication for ADHD and on melatonin for sleep issues. His renal scan was normal.

His genetic investigations include normal chromosomal analysis by microarray CGH. He was found to have a *de novo* nonsense pathogenic variant c.79G>T, p.Glu27* in *SOX4* gene in DDD study.

Patient 3

Patient 3 is a 13.5-year-old girl, born to healthy Caucasian parents. Pregnancy was complicated by gestational diabetes. Birth measurements were normal (all between 50th – 75th centile). At age 13 7/12 years her length was 152.5 cm and her weight was 45 kg. She had an OFC of 52.5 cm. Motor

development was normal, but speech was delayed. She is only able to speak a few words. She suffers from epilepsy (3-4 seizures per week), treated by Lamotrigin, and shows behavioral anomalies (esp. aggressiveness). Her facial phenotype is characterized by wide set eyes with epicanthus medialis and temporoparietal spared hair growth.

Table S2: Clinical features of patients with SOX4 variants.

	**Patient 1**	**Patient 2**	**Patient 3**
Gender	female	male	female
Age	12 years	12.5 years	13.7
Ancestry	German	Irish European	German
SOX4 variant	c.182G>A, p.Arg61Gln	c.79G>T, p.Glu27*	c.355C>T, p.His119Tyr
**Growth**			
pregnancy /gestational week	normal / 37+0	41	Gestational diabetes<br>37+0
Birth measurements			
Weight (g)	2420 (3rd-10th, -1.3 SD)	3810 (75th centile)	3045 (50th-75th, 0.2 SD)
Length (cm)	50 (25th-50th, 0.2 SD)	not available	50 (50th-75th, 0.2 SD)
OFC (cm)	33 (25th-50th, -0.6 SD)	not available	34.5 (50th-75th, 0.4 SD)
Measurements at last examination			
Height (cm)	144.5 (10th-25th, -0.9 SD)	140.5 (25th)	138 (P15)
Weight (kg)	32.8 (10th-25th)	40.5 (75th)	36.5 (P50)
OFC (cm)	52 (10th-25th, -1.1 SD)	52 (2nd)	54 (P79)
**Development**			
Speech delay	yes, delayed speech at 2,5 years, speaks in whole sentences at 13 years	Mild delay	Delayed speech,<br>Speaks only some words
Walking delay	yes, first steps at 19 months	no	no

Hypotonia	not described	no	yes
Intellectual and neurological delay	mild ID (IQ dropped from 79/81 to 58), learning difficulties requiring educational support	moderate learning disability,<br>Special needs in mainstream school 1:1 help	Learning disability,<br>Special school for handicapped children
Behavior anomalies	ADHD	ADHD, aggressive difficult behavior	Aggressive behavior
Seizures	no (single febrile seizure at 11 months)	no	3-4 seizures a week (medication: Lamotrigin)
**Clinical findings**			
Facial dysmorphism	deep-set eyes, mildly upslanting palpebral fissures, a broad philtrum, and a cupids bow	Low set, cup shaped ears, hypertelorism, epicanthal folds, thin upper lip, smooth philtrum	Wide set eyes with epicanthus medialis, temporoparietal spared hair growth
Hand and foot malformation	relatively short fingers with short terminal phalanges and 5th finger clinodactyly	Nail hypoplasia<br>Bilateral clinodactyly and hypoplasia involving 2nd and 5th digits<br>Bilateral hypoplasia of nails of 2nd and 5th toes	Clinodactyly
Other abnormal features	chronic constipation, hypothyroidism, unilateral doubled kidney, WPW syndrome, mild dilatation of the aorta ascendens, scoliosis	Widely spaced nipples	Muscular hypotonia<br>Atactic movement disorder, insomnia

Table S3: Experimental data of luciferase transactivation assays. Each experiment was conducted independently. Statistical analysis of each experiment was performed using Welch's unpaired t-test. SEM standard error of means.

POU3F2		+	-	+	+	+	+	+	+
SOX4		wt	wt	-	Arg61Gln	Glu27*	His119Tyr	Phe66Leu	Ala112Pro
#1	mean	0.564	0.032	0.009	0.014			0.035	0.035
	SEM	0.044	0.005	0.001	0.002			0.003	0.006
	p-value		0.007	0.006	0.006			0.007	0.007
#2	mean	2.532	0.250	0.052	0.041			0.139	0.116
	SEM	0.073	0.004	< 0.001	0.001			0.004	0.002
	p-value		0.001	0.001	0.001			0.001	0.001
#3	mean	1.203	0.142	0.034	0.031			0.035	0.127
	SEM	0.147	0.011	0.003	0.001			< 0.001	0.007
	p-value		0.019	0.015	0.015			0.015	0.018
#4	mean	1.273			0.036	0.047		0.049	0.046
	SEM	0.149			0.002	< 0.001		0.002	0.003
	p-value				0.014	0.014		0.015	0.015
#5	mean	2.909			0.030	0.073		0.043	0.080
	SEM	0.037			0.001	0.001		0.002	0.001
	p-value				< 0.001	< 0.001		< 0.001	< 0.001
#6	mean	1.287			0.025	0.030		0.035	0.027
	SEM	0.014			0.001	0.002		0.001	0.001
	p-value				< 0.001	< 0.001		< 0.001	< 0.001
#7	mean	2.242			0.022		0.141		
	SEM	0.070			< 0.001		0.036		
	p-value				0.001		0.001		
#8	mean	5.149					0.091		
	SEM	0.057					0.004		
	p-value						< 0.001		
#9	mean	1.588			0.020	0.019	0.020	0.018	0.015
	SEM	0.053			0.001	< 0.001	0.002	< 0.001	< 0.001
	p-value				0.001	0.001	0.001	0.001	0.001
#10	mean								
	SEM								
	p-value								

Table S4: Experimental data of protein-protein-interaction assay. Each experiment was conducted independently. Statistical analysis of each experiment was performed using Welch's unpaired t-test. SEM standard error of means.

POU3F2		+	+	+	pBiT2.1-N-FLAG-POU3F2	pBiT2.1-N-FLAG-empty
SOX4		wt	Arg61Gln	His119Tyr	pBiT1.1-C-myc-empty	pBiT1.1-C-myc-SOX4-wt
#1	mean	22996	55133	40687	1884	2756
	SEM	12214	3348	2039	78	101
	p-value		0.0054	0.0044	0.003	0.0029
#2	mean	176330	57592	29845	6361	5099
	SEM	0.073	3332	856	243	168
	p-value		0.0106	0.0065	0.0048	0.0048
#3	mean	489506	117168	107212	4802	6045
	SEM	26692	4438	4229	420	626
	p-value		0.0052	0.005	0.003	0.003
